# Neuroblastoma in the Elderly and SIADH: Case Report and Review of the Literature

**DOI:** 10.1155/2012/952645

**Published:** 2012-08-23

**Authors:** Micaela Pellegrino, Laura Gianotti, Sara Cassibba, Rodolfo Brizio, Alberto Terzi, Giorgio Borretta

**Affiliations:** ^1^Division of Endocrinology and Metabolism, Department of Internal Medicine, S. Croce e Carle Hospital, Via M. Coppino 26, 12100 Cuneo, Italy; ^2^Department of Pathology, S. Croce e Carle Hospital, Via M. Coppino 26, 12100 Cuneo, Italy; ^3^Thoracic Surgery Unit, S. Croce e Carle Hospital, Via M. Coppino 26, 12100 Cuneo, Italy

## Abstract

*Objective*. To report the rare case of a thymic neuroblastoma, in an elderly woman with SIADH at presentation. *Methods*. Clinical and biochemical data of the patient are presented and the pertinent literature is reviewed. *Results*. a 79-year-old woman was admitted into our department with worsening asthenia, severe hyponatremia (114.8 mEq/L), low plasma osmolarity (253 mEq/L), and inappropriate urinary sodium concentration (151 mEq/L). CT scan showed an a large solid inhomogeneous mass in the anterior mediastinum. ^18^F-FDG-PET/CT showed uptake in the mass. On continuous 3% hypertonic saline infusion, sodium gradually increased without achieving normal values. The patient underwent surgery, followed by full normalization of sodium levels. Tumor cells were positive for neuroendocrine markers. Thymic neuroblastoma with SIADH was diagnosed. *Conclusions*. Neuroblastoma is an extremely rare tumor in the elderly. Contrary to children and younger adults, neuroblastoma in older adults is typically localized in the anterior mediastinum and is often associated with SIADH. Moreover, it has mainly local aggressiveness in this age group, without metastatic spread; thus radical surgery achieves cure in most cases.

## 1. Introduction 

Neuroblastoma (NB) occurs commonly in children and is rare in adults. NB occurs in abdomen, thorax, and the head and neck regions in younger adults [1–3], whereas the anterior mediastinum is the most frequent localization in older adults (over 60 yrs) [3–7]. In the latter, only eight cases have been reported so far (Table 1).

Among the paraneoplastic syndromes occurring with NB, the syndrome of inappropriate secretion of antidiuretic hormone (SIADH) is frequently observed in the elderly, often asymptomatic, but never reported in children and only occasionally in young adults [6, 7].

The treatment of NB is surgical excision, although chemotherapy and radiotherapy can occasionally be used as adjuvant in some cases [2, 9, 10].

The prognosis is age-related. The disease is more aggressive and with a poorer outcome in younger adults, regardless of initial stage and age at the time of diagnosis. In contrast, the outcome is unknown in the elderly, where metastatic spread has been previously reported only twice [4, 8]. 

We report here a large thymic NB in an elderly woman, presenting as SIADH.

## 2. Case Report 

A 79-year-old woman was admitted to our department with progressive asthenia and severe hyponatremia (sodium 114.8 mEq/L). In the previous months, attention deficits and slight memory loss were reported. She was not taking any drug, beyond calcium channel blocker for mild hypertension. The patient and their relatives denied excessive water intake.

HCV-related liver disease had been diagnosed since 10 years.

At physical examination neither dehydration nor oedema were observed, BMI was 20.5 kg/m^2^, blood pressure was 145/90 mmHg, heart rate was regular, and lymph nodes were not palpable. 

Lab workup disclosed low-measured plasma osmolarity (253 mOsm/L) and urinary sodium concentration were 151 mEq/L. Other chemistries were within normal ranges, including blood urea nitrogen and creatinine, as well as thyroid and adrenal hormones, urinary catecholamines (norepinephrine, epinephrine, dopamine), and chromogranin A.

**Figure 1:**
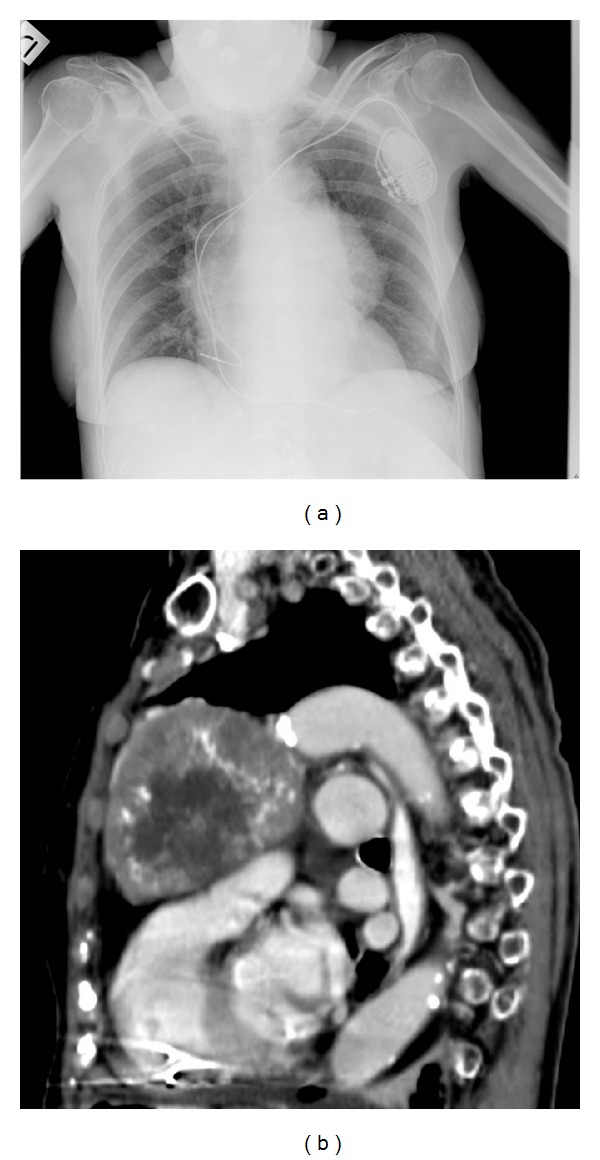
(a) Chest X-ray shows enlargement of the mediastinum. (b) Chest computed tomography image demonstrates a large inhomogeneous mass with calcifications in the anterior mediastinal compartment. The tumor is in close relationship with the ascending aorta and the common trunk of the pulmonary artery, with apparent preservation of a cleavage plane.

Chest X-rays revealed an abnormal enlargement in the mediastinum (Figure 1(a)). CT scan showed a large solid inhomogeneous mass originating in the anterior mediastinum and abutting into the left pleural space (about 10 × 8 × 7 cm, Figure 1(b)), as well as a nodular lesion in the 3rd liver segment (suspicious for hepatocarcinoma, HCC, already detected in a scan two years before), but was negative at the encephalic level. An ^18^F-FDG-PET/CT displayed positive uptake in the mass (SUVmax = 10).

On continuous 3% hypertonic saline infusion, sodium gradually increased (up to 128.5 mEq/L within 6 days), without achieving normal values.

Subsequently, the patient underwent surgery through an anterolateral thoracotomy that allowed the resection “en bloc” of a large mass originating in the anterior mediastinum, not infiltrating any intrathoracic structure except the pericardium. All the anterior mediastinal fatty tissue and thymus were excised as well, whereas the phrenic nerve was carefully dissected and spared.

Macroscopically, the tumor measured 12 × 8 × 6 cm and was surrounded by thymic fat. It was lobulated and well circumscribed. Its cut surface was gray, with areas of hemorrhage, and a thin fibrous peripheral capsule. 

**Figure 2:**
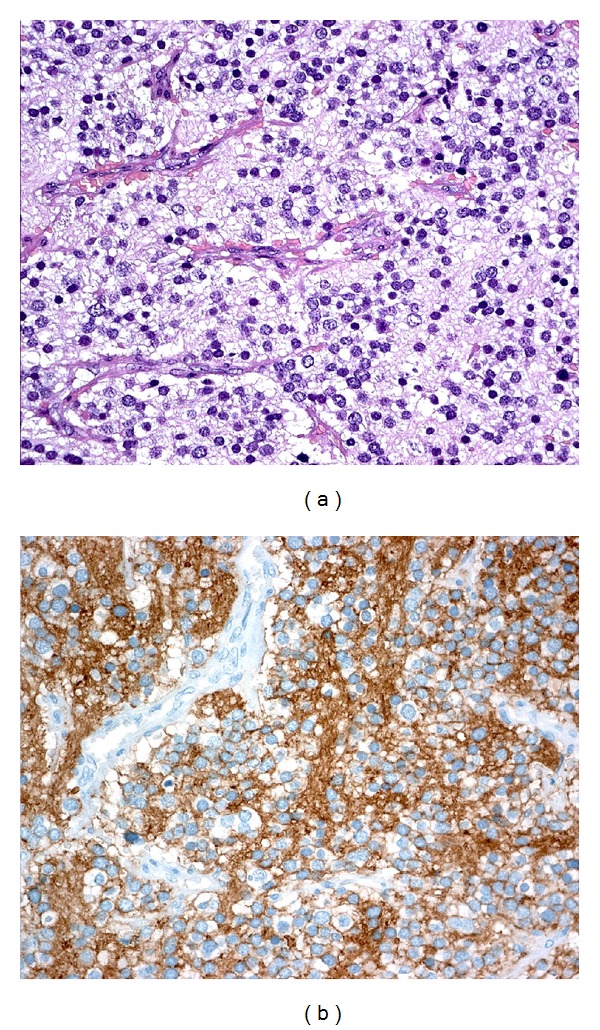
(a) The tumor shows a lobular architecture made of small uniform cells, with indistinct cell borders, hyperchromatic nuclei, fibrillary matrix material, and scattered large cells with eosinophilic cytoplasm. (b) The tumor is strongly positive for NSE immunostaining.

Microscopically, the tumor showed a lobular architecture made of small uniform cells, with indistinct cell borders, hyperchromatic nuclei, fibrillary matrix material, and scattered large cells with eosinophilic cytoplasm (Figure 2(a)). At immunohistochemistry tumor cells were positive for neuroendocrine markers: CD56, neuron specific enolase (NSE), synaptophysin, and chromogranin A (Figure 2(b)).

On the basis of these findings, a thymic neuroblastoma was diagnosed.

Sodium levels completely normalized within 2 days after surgery and patient's clinical conditions progressively improved.

At the 24-month followup, the patient is disease-free. Blood pressure levels are still controlled on the same calcium channel blocker. Sodium levels are still normal. 

## 3. Discussion

NB is a neural crest cell tumor. 

While NB is the most common extracranial solid tumor in children (accounting for about 8% of cancers in patients under 15 years), it is rare in adults (>30 yo, incidence: 0.2 cases per million inhabitants per year) [11] and extremely rare in the elderly (>60 yo) [3–7].

Mortality rate is reportedly low in children, whereas it is near 50% at 10 years after the diagnosis in adults, even in nonmetastatic tumors [12]. The mortality rate in elderly is unknown.

Usual NB sites are abdomen and posterior mediastinum in childhood, and abdomen, thorax, and the head and neck regions in young adults [1–3].

Only eight NBs have been previously reported in older adults (Table 1): six were in the anterior mediastinum (four out of these were intrathymic) and two were in the abdomen (one out of these was disseminated in the epidural spinal cord) [8].

NB is often symptomatic in young adults (pain and mass effects in 72% and 56%, resp.) but rarely associated to paraneoplastic syndromes. The literature reported SIADH in one sellar NB in a 29-yo patient and in nine esthesioneuroblastomas, an uncommon intranasal neuroendocrine tumor [13, 14]. In addition strong immunoreactivity for arginine vasopressin was reported in one very rare sinonasal teratocarcinosarcoma (mixed olfactory neuroblastoma-craniopharyngioma) in a 59-yo man [15].

On the other hand, SIADH was described in two elderly patients with thymic NB, with immunohistochemistry confirming the secretion of ADH [6, 7]. Therefore, paraneoplastic SIADH is more frequent in older than in younger adults bearing NB. Furthermore, other kinds of paraneoplastic syndrome have not been reported with NB in the elderly.

Beside NB, other thymic lesions can lead to SIADH. Four cases of large malignant thymoma, one case of thymic ganglioneuroblastoma, and one thymic carcinoma have been reported so far in adults (45–64 years old) [16–18].

Therefore, NB has to be considered the most frequent thymic tumor associated with SIADH in the elderly.

It is also interesting to highlight that different histological varieties of thymic tumors seem to have distinctive propensity to develop SIADH. To date, it is unclear whether this relationship could have anatomical-functional pathogenetic significance or is purely accidental. Nevertheless, thymic NB must be considered in the differential diagnosis of mediastinal tumors presenting with SIADH in older adults.

While in young adults one third of NBs are metastatic at diagnosis, in elderly people the metastatic dissemination is extremely rare, being previously reported only twice [4, 8]. This finding despite large size of NB at diagnosis in most cases, leads to consider NB as a low-grade tumor in older adults. 

So far, there are no approved standard guidelines for the treatment of NB. Currently, the treatment in adults is surgery when a nonmetastatic tumor is found. In locally invasive tumors, surgery is followed by radiotherapy, while in patients with metastatic disease a multimodal treatment is indicated including surgery, radiotherapy, and chemotherapy [2, 11].

In children below 1, year the prognosis of NB is good. In adolescents or young adults the ultimate outcome is generally poor, regardless of the initial disease stage and age at diagnosis [11]. Owing to the rarity of NB in adults, limited data are available on its prognosis. A poor outcome was frequent in the cases previously reported, mostly diagnosed in advanced stage.

The literature data about prognosis in elderly patients are scanty. Argani et al. reported one patient who died of progressive disease; another patient was disease-free at 18 months after surgery and another one died of unrelated causes. In 2010 Ohtaki et al. reported a case of a 64-yo man who was alive 10 months after the resection of lung metastasis that appeared 7 month after surgery. Among the two patients with an abdominal NB, one is reportedly disease-free at 108 months after surgery [2] and the second died owing to progressive disease [8].

We report the fifth case of a thymic NB, the largest one described in the literature, which was associated to SIADH in an elderly woman. In contrast to other reports, our patient was symptomatic with severe asthenia and memory impairment. Subsequently, a large mediastinal mass was found without any mass effect despite its size. Histological examination demonstrated the neuroendocrine origin of the tumor.

We cannot rule out the possibility of other sources of SIADH, because immunohistochemical demonstration of ADH could not be established on the surgical specimens due to antibodies unavailability. An impaired venous return caused by the huge mass could have been hypothesized, but the patient did not show any clinical sign of mediastinal syndrome. Iatrogenic causes of SIADH and excessive water intake were excluded as well.

Ectopic production of ADH by the hepatic nodule shown by TC could be also considered as a possible cause, but it seems unlikely because sodium levels normalized after surgery.

Furthermore, the positivity of NSE and chromogranin A, as well as the thymic localization of the mass (all NBs with ectopic ADH secretion described so far were intrathymic), make likely the paraneoplastic origin of SIADH.

In our patient, the absence of metastatic dissemination (the hepatic nodule was regarded as HCC in HCV-related liver disease) supports an indolent course of the disease and points to a good prognosis, despite pericardial invasion and large size at diagnosis. Our patient is alive and disease free at 24 months after surgery.

In conclusion, NB is an extremely rare tumor in the elderly often incidentally diagnosed, though it can be very large. In contrast to those described in children and younger adults, NB in older adults is typically localized in the anterior mediastinum and is often associated to SIADH. Therefore, in this age group, NB has mainly local aggressiveness, without metastatic behaviour, and radical surgery is curative in most cases. Thus, thymic NB should be kept in mind during the differential diagnosis of hyponatremia in the elderly, a common electrolyte disorder in this age group.

## Figures and Tables

**Table 1:** Clinical characteristics of the literature patients.

Patient no.	Primary site	Age (yr)/sex	Metastases	SIADH	Reference no.
1	Anterior mediastinum	80/F	NR	−	[5]
2	Thymus	Elderly 1/NR	NR	+	[6]
3	Thymus	Elderly 2/NR	NR	−	[6]
4	Thymus	Elderly 3/NR	NR	−	[6]
5	Thymus	60/M	−	+	[7]
6	Adrenal	69/M	−	NR	[2]
7	Adrenal	67/F	SK, paravertebral soft tissues	−	[8]
8	Superior mediastinum	64/M	LN	−	[4]

F: female, M: male, SK: skeleton, LN: lymph nodes, and NR: not reported.

## Abbreviations

HCC: Hepatocarcinoma
NB: Neuroblastoma
SIADH: Syndrome of inappropriate secretion of antidiuretic hormone
NSE: Neuron specific enolase.
